# Antimicrobial Susceptibility Testing for Three *Malassezia* Species

**DOI:** 10.1128/spectrum.05076-22

**Published:** 2023-06-13

**Authors:** Brooke Rathie, Bart Theelen, Martin Laurence, Rebecca S. Shapiro

**Affiliations:** a Department of Molecular and Cellular Biology, University of Guelph, Guelph, Ontario, Canada; b Westerdijk Fungal Biodiversity Institute, Utrecht, The Netherlands; c Malassezia Foundation, Montreal, Quebec, Canada; Universidade de Sao Paulo

**Keywords:** antifungals, antimicrobial resistance, Malassezia

## Abstract

The *Malassezia* genus comprises lipid-dependent yeasts that have long been associated with common skin diseases, and have recently been linked with Crohn’s disease and certain cancers. Understanding *Malassezia* susceptibility to diverse antimicrobial agents is crucial for identifying effective antifungal therapies. Here, we tested the efficacy of isavuconazole, itraconazole, terbinafine, and artemisinin against three *Malassezia* species: *M. restricta*, *M. slooffiae,* and *M. sympodialis*. Using broth microdilution, we found antifungal properties for the two previously unstudied antimicrobials: isavuconazole and artemisinin. Overall, all *Malassezia* species were particularly susceptible to itraconazole, with a MIC range from 0.007 to 0.110 μg/mL.

**IMPORTANCE** The *Malassezia* genus is known to be involved in a variety of skin conditions and has recently been associated with diseases such as Crohn’s disease, pancreatic ductal carcinoma, and breast cancer. This work was completed to assess susceptibility to a variety of antimicrobial drugs on three *Malassezia* species, in particular *Malassezia restricta*, which is an abundant *Malassezia* species both on human skin and internal organs and has been implicated in Crohn’s disease. We tested two previously unstudied drugs and developed a new testing method to overcome current limitations for measuring growth inhibition of slow-growing *Malassezia* strains.

## OBSERVATION

The most prevalent fungal genus of the human skin microbiome is *Malassezia* (1). While these fungi are a normal part of the human skin flora, they are also known agents in skin disorders such as dandruff and seborrheic dermatitis, as well as systemic infections in immunocompromised individuals (2, 3). Treatment for dermatologic disorders associated with *Malassezia* includes ketoconazole and itraconazole (4). It has been convincingly shown that *Malassezia* is highly prevalent in human mucosal surfaces such as the mouth, gut, nose, pancreas, and vagina (5–7). *Malassezia restricta* has been most frequently found within internal organs (5, 6). Recent studies have implicated *Malassezia* in Crohn’s disease (8), pancreatic ductal carcinoma (9), and breast cancer (10), raising the possibility that reducing or eliminating *Malassezia* from affected organs might improve clinical outcomes of these diseases. However, systemic antifungal treatment of *Malassezia* remains unstudied clinically.

The aim of this study was to evaluate the *in vitro* activity of diverse systemic antimicrobial agents against three *Malassezia* species: *M. restricta*, *M. sympodialis*, and *M. slooffiae.* To our knowledge, this is the first study to examine the effects of isavuconazole and artemisinin on the *Malassezia* genus. We also demonstrated a simple and inexpensive assay that may be useful for future testing of antimicrobial agents against *Malassezia*.

We studied the susceptibility of four antimicrobials: itraconazole, isavuconazole, terbinafine, and artemisinin. Itraconazole and isavuconazole are both triazoles which inhibit lanosterol 14α-demethylase, an enzyme necessary for the biosynthesis of ergosterol, the critical sterol of the fungal cell membrane. Terbinafine is an allylamine that inhibits squalene epoxidase, an enzyme which catalyzes the conversion of squalene to lanosterol in the ergosterol synthesis pathway. Artemisinin is an antimalarial drug which has also been reported to have fungistatic activity, but has yet to be tested against *Malassezia* species (11–13). The mechanisms of action of artemisinin against fungi are still unknown. However, there has been research showing artemisinin influences Ca^2+^ ATPases in Plasmodium falciparum, Saccharomyces cerevisiae, and Candida glabrata (13).

Antifungal susceptibility testing of *Malassezia* has been limited by the inability to reliably utilize conventional yeast microbiology protocols (14). Comparisons between MIC experimental assays have proven to lack congruence (15, 16). Past studies have exploited large strain numbers to determine a general trend for *Malassezia* susceptibility to antifungals, including itraconazole among a variety of azoles, as well as terbinafine and amphotericin B (15–18). While there are multiple studies which have examined faster-growing *Malassezia* species MICs, few have examined the slow-growing *Malassezia* species such as *M. restricta* (16, 18).

Turbidity readings of *Malassezia* can underestimate growth and are unreliable due to the fungi’s tendency to clump. Past studies have used a colorimetric MIC method to overcome this issue for the *Malassezia* genus, utilizing resazurin to measure cell metabolism during or after antifungal treatment (16, 19, 20). Here, resazurin-based methods utilized in past studies to measure growth inhibition were built upon to develop a simple and effective process to measure *Malassezia* susceptibility *in vitro*, including for the slower-growing *M. restricta.*

**FIG 1:**
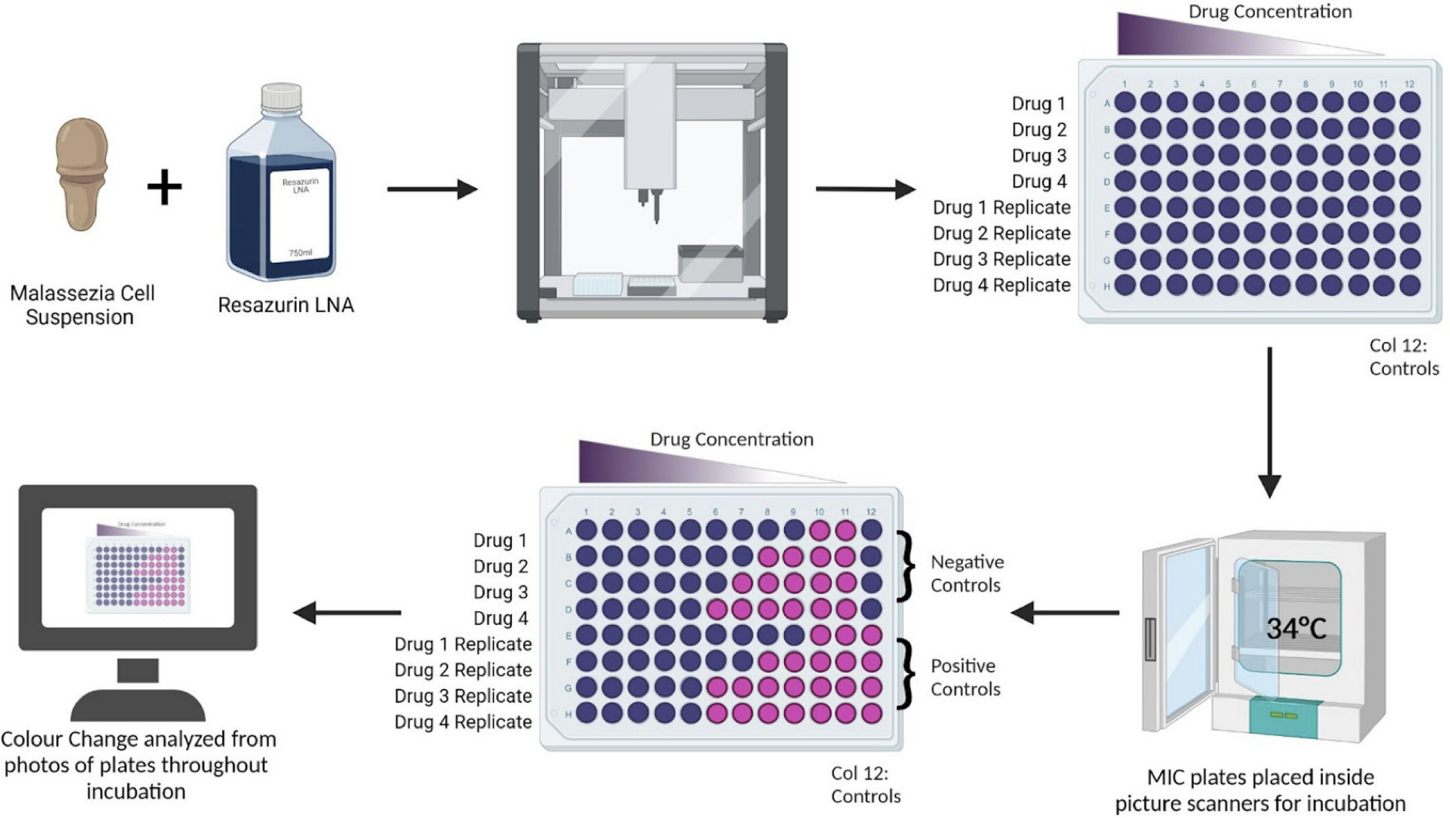
Workflow overview for MIC assay performed.

We aimed to develop a novel experimental setup that allows for kinetic monitoring of *Malassezia* growth in a high-throughput manner, while limiting the need for specialized and costly lab equipment (Fig. 1). We used flatbed scanners housed within a large incubator to automatically photograph *Malassezia* cultures in 96-well microtiter plates every 2 h. With this setup, *Malassezia* MIC analyses were conducted using standard antimicrobial drug dilutions (Fig. 1). By extracting the average brightness of each well, we were able to measure and quantify cell metabolism using resazurin dye as a growth indicator.

This experimental protocol helped provide an unbiased and accurate relative MIC value for each drug condition tested. We used this platform to study the effects of itraconazole, isavuconazole, artemisinin, and terbinafine on *Malassezia* species. To assess differences in drug susceptibility between strains, we established drug resistance to be a difference in the MIC value of two dilution steps or more.

Overall, clinical isolates of *M. sloofiae* did not exhibit susceptibility differences between strains. MIC50s of *M. sloofiae* were within one dilution step and exhibited the highest susceptibility to itraconazole (Table 1). *M. sympodialis* was highly susceptible to itraconazole with an MIC50 of 0.007 μg/mL and overall drug susceptibility similar to *M. restricta* CBS7877 (Table 1).

**TABLE 1:** MIC50 values for *Malassezia* strains^*a*^

Strain no.	Species	Itraconazole (μg/mL)	Isavuconazole (μg/mL)	Terbinafine (μg/mL)	Artemisinin (μg/mL)
CBS7877 (*n* = 4)	*M. restricta*	0.007	0.017	0.091	0.088
BTH004 (*n* = 4)	*M. restricta*	0.055	0.273	0.091	0.176
KCTC27527 (*n* = 4)	*M. restricta*	0.007	0.017	0.046	0.176
KCTC27524 (*n* = 5)	*M. restricta*	0.007	0.034	0.046	0.353
BTH006 (*n* = 5)	*M. slooffiae*	0.028	0.068	0.046	0.176
BTH007 (*n* = 4)	*M. slooffiae*	0.014	0.068	0.046	0.088
BTH016 (*n* = 5)	*M. slooffiae*	0.028	0.068	0.046	0.088
BTH012 (*n* = 4)	*M. sympodialis*	0.007	0.068	0.091	0.044

a MIC50 is the first concentration for which growth has a relative growth of 50% or below. These values were calculated from the ratio of drug-treated cells to the growth of cells without drug treatment.

**FIG 2:**
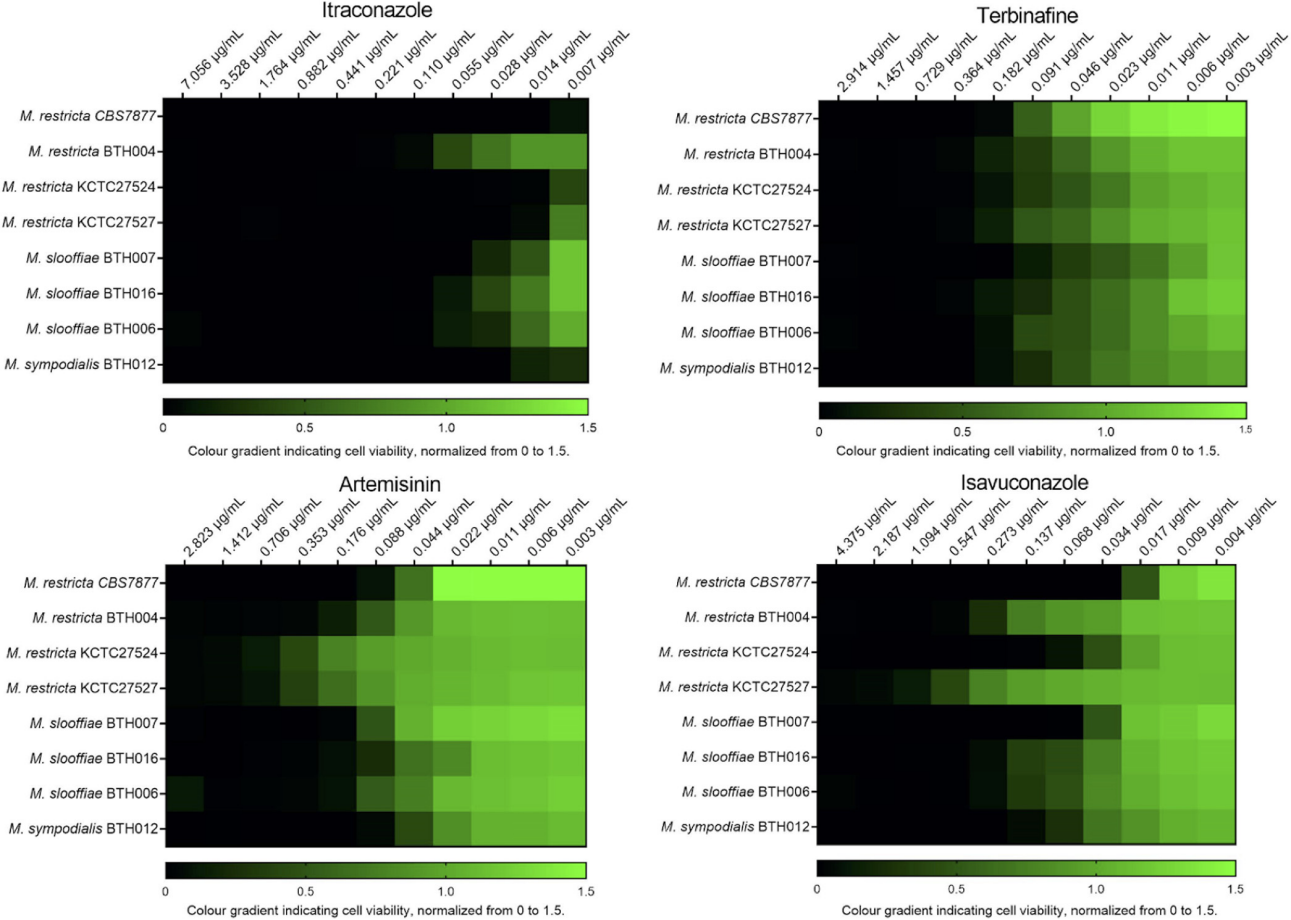
Heat map depiction of MIC values from various *Malassezia* strains. Fungi viability values were created through computational quantification of luminescence, values were normalized from 0 to 1.5 with 1.5 being the average growth of the nontreated control wells.

We examined the *M. restricta* type strain CBS7877 and three *M. restricta* clinical isolates. Of the four antimicrobials, itraconazole was the most effective drug with a MIC range of 0.007 to 0.110 μg/mL (Fig. 2). The clinical strain BTH004 exhibited reduced susceptibility to itraconazole relative to the wild-type strain, with a MIC50 of 0.055 μg/mL, three or four dilution steps higher (Table 1). Isavuconazole had a MIC range of 0.055 to 0.007 μg/mL (Fig. 2). Isavuconazole resistance was observed in KCTC27524 and BTH004 which was four dilution steps more resistant than CBS7877. Terbinafine had higher MICs and were all within one dilution step: 0.182 to 0.091 μg/mL. Artemisinin antimicrobial effects were similar to terbinafine with the exception of KCTC27524 and KCTC27527 which exhibited resistance against artemisinin (Fig. 2).

Utilizing a novel experimental technique, we were able to investigate the effects of four systemic antimicrobials on clinical and type strains of *Malassezia* fungi. This new method avoids optical density (OD) measurement inaccuracies due to *Malassezia* clumping (observed in laboratory practice; data not provided). Overall, the antifungal itraconazole showed the highest potency against the diverse *Malassezia* strains studied. Although artemisinin was less effective than the triazoles, it is currently more affordable than other antifungals tested and has been used in clinical applications with doses of up to 500 mg/day with no major side effects (21). Therefore, artemisinin would be a good *Malassezia* antifungal candidate, as the dose necessary for treatment is likely not to elicit major side effects.

Our results agree with current literature that has suggested itraconazole to be an effective drug against the *Malassezia* genus (16, 17). Itraconazole has been found to have a MIC range from 0.01 to 0.03 μg/mL. Past research into the MIC of terbinafine against *Malassezia* ranged from 0.03 to >16 μg/mL (14, 15, 17). Lack of standardized MIC testing guidelines make these results difficult to compare. Additionally, even within a single study MICs between clinical strains tested can vary significantly (17).

For the first time, we tested the antifungal effects of both isavuconazole and artemisinin against *Malassezia* and showed they are effective agents against *Malassezia* species. Future investigation into the effects of antifungals for treatment of Malassezia-associated systemic diseases may provide improved outcomes for patients. We hope to have provided some preliminary guidance for methodologies of testing *Malassezia* *in vitro* with antifungals as well as information regarding future options for *Malassezia* treatments.

*Malassezia* strains tested include *M. restricta* (CBS7877), *Malassezia* gifted from Won Hee Jung at the Chung-Ang University, Korea; *M. restricta* (KCTC27524, KCTC27527) and *Malassezia* gifted from Bart Theelen from the Westerdijk Fungal Biodiversity Institute; *M. restricta* (BTH004), *M. slooffiae* (BTH006, BTH007), and *M. sympodialis* (BTH012, BTH016). KCTC27524 and KCTC27527 were isolated from Korean patients with seborrheic dermatitis of the scalp. All *Malassezia* strains were cultured and maintained on Leeming-Notman agar (LNA). Species identification was performed through Sanger sequencing internal transcribed spacer (ITS) regions and then analyzed using NCBI-BLAST. To achieve a uniform inoculum, culture was suspended in bead bashing tubes and vortexed for 5 s. Inoculum suspended in saline was then diluted to an OD600 of 0.3.

Liquid LNA-MIC media was prepared by mixing 7.5 g bacteriological grade peptone (Oxoid LP0037), 6 g ox bile (Hardy Diagnostics C6511), 3.75 g glucose, 0.075 g yeast extract, 0.375 g glycerol monostearate (Axenic 123-94-4), 0.375 g chloramphenicol (Biobasic CB0118), and 3 g of cycloheximide (Cayman Chemicals 14126), in 750 mL of demineralized water, heated to 50°C to 60°C; then adding 375 μL of glycerol, 750 μL of Tween 60. Media was heated to 95°C before allowing it to cool. Once cooled to 42°C, 0.03 g of resazurin was added. Media was sterilized through a 0.045 μm filter and stored at room temperature in a dark space for up to a week (alternatively up to 2 weeks at 4°C).

*Malassezia* activity was quantified through change of color of the metabolic indicator dye resazurin. Antimicrobials tested (itraconazole, isavuconazole, artemisinin, and terbinafine) all were dissolved in DMSO at a concentration of 2 mM and stored at –20C. All protocols were performed in an Opentrons-OT2 liquid handler. In a 96-well flat bottom untreated NEST plate, each well was filled with 125 μL of LNA-MIC media. Drugs were then added to column 1 to a concentration of 20 μM and serially diluted across the plate until column 11. Fungal inoculum was added to 12 mL of LNA-MIC and 155 μL of inoculated media was added to each well. All wells are inoculated with fungi except for four negative-control wells in column 12. The other four wells of column 12 were utilized as positive-control wells. Continuous monitoring allowed equal inoculation of each strain of fungi, regardless of species, as endpoint times were readily altered to adjust for differences in growth rate and metabolism. *Malassezia* strain was separately tested against all four drugs in duplicate in each 96-well microtiter plate.

Plates were covered with a gas-permeable membrane (Diversified Biotech, BEM-1) and incubated at 34°C for 80 h on a flatbed scanner (Canon CanoScan LiDE 300). Scans were performed automatically every 2 h, and saved as 200 dpi color JPEG files. All wells start dark blue, and slowly turn pink as resazurin is degraded, measuring the metabolic activity of *Malassezia* cells within the well. MIC results were recorded when the positive-control wells fully flipped to pink (between 50 and 80 h). Quantification of luminance was performed using a custom Python script (tlscan2.py) which calculates the average brightness of each well using the Python Image Library (PIL). Briefly, each well is cropped out of the JPEG image using “.crop(),” then reduced to a single pixel using “.resize([1,1])” (this calculates the average brightness), then converted to grayscale using “.convert(‘L’),” and finally extracted as a integer in the range 0 to 255 using “.getpixel((0,0))” (0 = darkest, 255 = brightest, encoded using sRGB gamma compression). Data obtained went through blank reduction and normalized to the control before being averaged. Averaged MIC data were analyzed to obtain the recorded MIC values.
